# DiSMVC: a multi-view graph collaborative learning framework for measuring disease similarity

**DOI:** 10.1093/bioinformatics/btae306

**Published:** 2024-05-07

**Authors:** Hang Wei, Lin Gao, Shuai Wu, Yina Jiang, Bin Liu

**Affiliations:** School of Computer Science and Technology, Xidian University, Xi’an, Shaanxi 710126, China; School of Computer Science and Technology, Xidian University, Xi’an, Shaanxi 710126, China; School of Computer Science and Technology, Xidian University, Xi’an, Shaanxi 710126, China; Department of Basic Medicine, Shaanxi University of Chinese Medicine, Xianyang, Shaanxi 712046, China; Faculty of Engineering, Shenzhen MSU-BIT University, Shenzhen, Guangdong 518172, China; School of Computer Science and Technology, Beijing Institute of Technology, Beijing, 100081, China

## Abstract

**Motivation:**

Exploring potential associations between diseases can help in understanding pathological mechanisms of diseases and facilitating the discovery of candidate biomarkers and drug targets, thereby promoting disease diagnosis and treatment. Some computational methods have been proposed for measuring disease similarity. However, these methods describe diseases without considering their latent multi-molecule regulation and valuable supervision signal, resulting in limited biological interpretability and efficiency to capture association patterns.

**Results:**

In this study, we propose a new computational method named DiSMVC. Different from existing predictors, DiSMVC designs a supervised graph collaborative framework to measure disease similarity. Multiple bio-entity associations related to genes and miRNAs are integrated via cross-view graph contrastive learning to extract informative disease representation, and then association pattern joint learning is implemented to compute disease similarity by incorporating phenotype-annotated disease associations. The experimental results show that DiSMVC can draw discriminative characteristics for disease pairs, and outperform other state-of-the-art methods. As a result, DiSMVC is a promising method for predicting disease associations with molecular interpretability.

**Availability and implementation:**

Datasets and source codes are available at https://github.com/Biohang/DiSMVC.

## 1 Introduction

Uncovering disease associations is of great significance in biomedical research. Similar diseases tend to exhibit analogous clinical manifestations or stem from similar molecular mechanisms. Therefore, measuring disease similarity cannot only unravel disease pathological mechanisms but also foster advancements in disease diagnosis (Xiang *et al.* 2022), particularly in the identification of downstream disease-related genes (Le 2020, Cao *et al.* 2022), noncoding RNAs (Wei *et al.* 2021, Dai *et al.* 2022, Sun *et al.* 2022, Yan *et al.* 2022, Zhang and Liu 2022), and other biomarkers (Wang *et al.* 2023d, Cao *et al.* 2024). Moreover, such research contributes to therapeutic interventions, facilitating drug discovery (Wang *et al.* 2023c), and drug repurposing (Bang *et al.* 2023) by discovering analogous treatment targets across different diseases.

With the in-depth study of diseases, more and more biomedical data sources are collected. For examples, the semantic terminology systems including International Classification of Diseases (ICD) (Giles and Sander 2012), Disease Ontology (DO) (Bello *et al.* 2018), and Medical Subject Headings (MeSH) (Dhammi and Kumar 2014) have been constructed, where diseases are organized by hierarchical tree structures. In addition, a large number of diseases with phenotypic annotations are recorded in Human Phenotype Ontology (HPO) (Gargano *et al.* 2024), Orphanet (Gomez-Paramio *et al.* 2011), and PhenoDis (Adler *et al.* 2018). To reveal the underlying pathological mechanism at bio-molecule level, different types of biological databases such as DisGeNET (Piñero *et al.* 2017), dbSNP (Sherry *et al.* 2001), MNDR (Ning *et al.* 2021), and Reactome (Fabregat *et al.* 2018) have been established, providing experimentally validated associations between diseases and genes, SNPs, ncRNAs, and pathways, respectively.

The accumulation of biomedical data makes it possible to measure disease similarity by computational methods. There are three main categories of methods semantic-based, phenotype-based, and molecule-based. The semantic-based methods measure disease similarity by considering the path length or information content of different terms in the hierarchical network of diseases (Li *et al.* 2003, Wang *et al.* 2007, Yu *et al.* 2015, Feng *et al.* 2022, Ai *et al.* 2023), where their corresponding computational tools have been widely used to construct disease similarity networks (Ma and Jiang 2020, Wang *et al.* 2023e, Zhu *et al.* 2023). The phenotype-based methods mainly depend on the common phenotypic characteristics of diseases, where similar diseases may share more phenotype annotations (Deng *et al.* 2015, Peng *et al.* 2018, Wang *et al.* 2023b). Compared to the semantic and phenotype-based methods, the molecule-based methods can explore potential disease relationships from micro-level and achieve superior performance, where diseases can be described by their associated molecules via graph representation learning techniques and aggregation strategies (Cheng *et al.* 2014, Yang *et al.* 2021, Chen *et al.* 2022, Zeng *et al.* 2022a). Despite the significant advances in computing disease similarity, there are still two main issues that need to be addressed: (i) the occurrence and development of disease is an extremely complicated process, involving the interaction and regulation of different bio-molecules. Most molecule-based methods represent diseases solely at the genetic level, failing to capture sufficient semantic information; (ii) existing studies measure disease similarity in an unsupervised manner and ignore the valuable priori disease association knowledge, which may cause inadequate pattern learning and limited prediction efficiency (Zeng *et al.* 2022b).

This study is initiated in an attempt to overcome these problems by designing a novel computational method called DiSMVC to measure disease similarity. DiSMVC is a supervised graph collaborative framework including two major modules cross-view graph contrastive learning and association pattern joint learning. The former aims to enrich disease representation by considering their underlying molecular mechanism from both genetic and transcriptional views, while the latter captures deep association patterns by incorporating phenotypically interpretable multimorbidities in a supervised manner. Experimental results indicated that DiSMVC can identify molecularly interpretable similar diseases, and the synergies gained from DiSMVC contributed to its superior performance in measuring disease similarity.

## 2 Materials and methods

### 2.1 Datasets

To enrich disease description and explore their underlying molecular mechanism, five bio-entity networks including gene interaction network, miRNA similarity network, gene–miRNA interaction network, gene–disease, and miRNA–disease association networks are constructed and integrated into the proposed graph collaborative framework.

The gene interaction network $G^{g}$ is downloaded from HumanNet-FN (Kim *et al.* 2022), collecting the co-functional gene links. The miRNA similarity network $G^{m}$ is constructed by Gaussian Interaction Profile (GIP) kernel similarity measurement, where the interaction profiles are expressed as the association vector between miRNAs and diseases. The network $G^{gm}$ collects the gene–miRNA interactions with Transcriptome Dysregulation Measure Score (TDMDScore) >1, derived from ENCORI (Li *et al.* 2014). All the gene–disease associations collected in DisGeNET v7.0 (Piñero *et al.* 2017) and the experimentally validated miRNA–disease associations collected in RNADisease (Chen *et al.* 2023a) are downloaded to construct two heterogeneous networks, $G^{dg}$ and $G^{dm}$, respectively. In these networks, the genes and miRNAs excluded from $G^{g}$ and $G^{m}$ are filtered out. The specific statistical information of above constructed networks is listed in Table 1.

**Table 1.** The statistical information of different datasets.

**Dataset** ^a^	No. of nodes	No. of edges	Source
$G^{g}$	17247 genes	371501	HumanNet
$G^{m}$	4798 miRNAs	126264	RNADisease
$G^{gm}$	17247 genes, 4798 miRNAs	80114	ENCORI
$G^{dg}$	30170 diseases, 17247 genes	1030082	DisGeNET
$G^{dm}$	30170 diseases, 4798 miRNAs	355196	RNADisease

a $G^{m}$ was constructed by Gaussian Interaction Profile kernel similarity measurement based on experimentally validated miRNA–disease associations.

We detect the disease association pattern in a supervised manner, the prior disease associations are downloaded from the previous study (Dong *et al.* 2021), which analyzed the multimorbidities among common diseases in the UK Biobank. After unifying disease identifiers across multiple datasets and filtering out disease associations with criteria of RR value < 15 following the study (Dong *et al.* 2021), 3091 high-quality disease associations supported with phenotypic interpretability are collected to construct $S_{\mathrm{benchmark}}^{+}$ for training process.

To increase the data coverage and comprehensively evaluate the performance of different methods, the phenotypically and genetically interpretable multimorbidities in the study (Dong *et al.* 2021), the verified highly similar disease pairs predicted by the study (Mathur and Dinakarpandian 2012), and the validated disease pairs obtained based on the electronic health records (EHR) of the US population (Pakhomov *et al.* 2010) are integrated to construct testing positive set $S_{\mathrm{independent}}^{+}$. The dataset can be represented as:


(1) $$\left\{\begin{array}{c} \;S_{\mathrm{benchmark}}=S_{\mathrm{benchmark}}^{+}\;⋃\;S_{\mathrm{benchmark}}^{-}\\ {\;S}_{\mathrm{independent}}=S_{\mathrm{independent}}^{+}\;⋃\;S_{\mathrm{independent}}^{-} \end{array}\right.$$

where $S_{\mathrm{benchmark}}\cap S_{\mathrm{independent}}=\emptyset$, $S_{\mathrm{benchmark}}^{+}$ includes 3091 training positive pairs while $S_{\mathrm{independent}}^{+}$ includes 665 testing positive pairs. $S_{\mathrm{benchmark}}^{-}$ and $S_{\mathrm{independent}}^{-}$ are constructed by randomly selected negative pairs with the same number of positive set. The datasets can be downloaded from https://github.com/Biohang/DiSMVC/tree/main/Dataset.

### 2.2 Method overview

**Figure 1.**
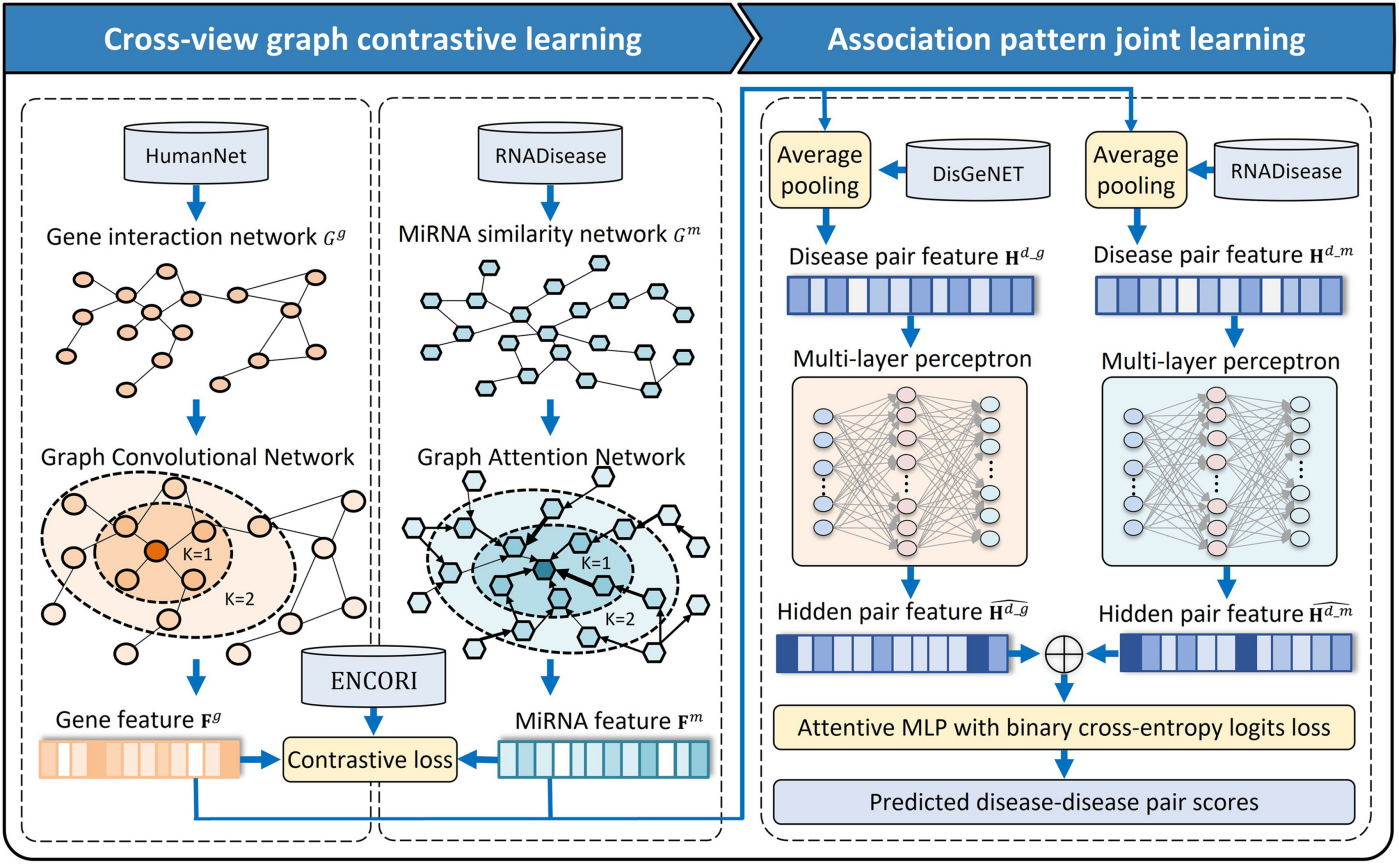
The framework of DiSMVC. There are two main steps: (i) Cross-view graph contrastive learning. Gene interaction network and miRNA similarity network are constructed, based on that node features are extracted by considering their proximity structures via different graph representation algorithms. Graph contrastive learning is implemented to further refine the hidden features of genes and miRNAs. (ii) Association pattern joint learning. Average pooling and concatenate strategies are applied to obtain initial disease pair features based on various priori bio-entity networks. Multi-layer perceptron models are jointly learned to detect hidden association patterns and predict disease similarity scores.

The framework of DiSMVC contains two main modules (cross-view graph contrastive learning and association pattern joint learning). As shown in Fig. 1, the former module is designed to extract the features of disease-related molecules via horizontally collaborative learning across different networks, and the latter module aims to detect association patterns between diseases via vertical joint learning. These two modules will be introduced in the following sections.

### 2.3 Cross-view graph contrastive learning

#### 2.3.1 Homogenous bio-network construction

Because the deregulation of similar genes or miRNAs may tend to be closely related to the development of similar diseases, it is reasonable to detect disease association patterns from molecular mechanisms. In this study, two homogeneous bio-networks including gene interaction network $G^{g}$ and miRNA similarity network $G^{m}\;$are constructed. The weight score $w_{i,j}$ between genes $g_{i}$ and $g_{j}$ in $G^{g}$ is calculated by:


(2) $$w_{i,j}=\frac{l_{i,j}-l_{\min}}{l_{\max}-l_{\min}}$$

where $l_{i,j}$ represents the log likelihood similarity score obtained from HumanNet (Kim *et al.* 2022), and $l_{\max}$ and $l_{\min}$ are the maximum and minimum log likelihood similarity scores between genes. The network $G^{m}$ mainly collects the functionally similar miRNAs, where the similarity score $s_{i,j}$ between miRNAs $m_{i}$ and $m_{j}$ is computed via the Gaussian Interaction Profile (GIP) kernel similarity measurement:


(3) $$\{\begin{array}{c} s_{i,j}=\text{exp}(-\gamma {\left\|A_{i}^{dm}-A_{j}^{dm}\right\|}^{2})\\ \gamma =\gamma \prime /(\frac{1}{n}{\sum_{p=1}^{n}\left\|A_{p}^{dm}\right\|}^{2}) \end{array}$$

where $A^{dm}\in R^{30170*4798}$ is an adjacency matrix of miRNA–disease association network $G^{dm}$. $A_{i}^{dm}$and $A_{j}^{dm}$ is the *i*th and *j*th row in matrix **A**^*dm*^, representing the interaction profiles of miRNA $m_{i}$ and $m_{j}$ with different diseases, respectively. n is the number of miRNAs, while $\gamma^{'}$ denotes the original kernel bandwidth and is defined as 1 following the previous study (Wei and Liu 2020, Wang *et al.* 2023a). To ensure the high quality of miRNA similarity network, the edges with similarity score > 0.8 are retained in $G^{m}$.

#### 2.3.2 Intra-view graph representation

Graph representation is designed to encode and extract meaningful embeddings preserving intricate structural properties from graph-structured data, and has been successfully applied in various bioinformatics tasks, such as circRNA-disease association detection (Wang *et al.* 2020, Chen *et al.* 2024, Niu *et al.* 2024), drug–target binding affinity prediction (Öztürk *et al.* 2018), cell–cell interaction identification (Yang *et al.* 2023). Inspired by the powerful ability of graph neural networks to uncover hidden semantic knowledge from bio-entity networks (Zhou *et al.* 2020, Yi *et al.* 2022), we adopt two graph encoders including Graph Convolutional Networks (GCN) (Kipf and Welling 2017) and Graph Attention Networks (GAT) (Veličković *et al.* 2018) for exploring the latent interaction pattern from each homogeneous bio-network.

For the gene interaction network $G^{g}$, graph encoder with GCN is constructed. GCN utilizes convolutional operation and message passing mechanism on network to learn and update node embeddings by considering the local and global neighborhood structure. For the *l + 1*th network layer, the gene feature matrix **F**^*g, l+1*^ can be obtained by:


(4) $$F^{g,l+1}=\sigma (D^{-\frac{1}{2}}\hat{A^{g}}D^{-\frac{1}{2}}F^{g,l}W^{l})$$

where $\hat{A^{g}}=A^{g}+I^{g}$ denotes the adjacency matrix of gene interaction network $G^{g}$ with inserted self-loops, the elements in $A^{g}\in R^{17247*17247}$ are the weight scores [cf Equation (2)]. **D** is a diagonal degree matrix calculated by$D_{ii}=\sum_{j=1}^{n}A_{ij}^{g}$, where *n* denotes the number of genes in $G^{g}$. $W^{l}$ is the learnable weight matrix from the *l*th layer, $F^{g,0}$ denotes the initial feature matrix of genes constructed by one-hot encoding, and σ represents the activation function defined as LeakyReLU.

To reduce noise interference in miRNA similarity network $G^{m}$, graph encoder with GAT is constructed for extracting miRNA features. Different from GCN, GAT extracts node features via aggregating their neighborhoods’ information with different attention coefficients and can apply to inductive as well as transductive problems (Yan *et al.* 2023, Zhang *et al.* 2024). For the *l + 1*th network layer, the feature vector $f_{i}^{m,l+1}$ of miRNA $m_{i}$ can be obtained by three main steps. Firstly, the attention coefficient $e_{ij}$ representing the importance of miRNA $m_{j}$ to $m_{i}$ is calculated by:


(5) $$e_{ij}=\mathrm{LeakyReLU}(a^{T}(W^{l}f_{i}^{m,l}∥W^{l}f_{j}^{m,l}))$$

where a is a learnable attention parameter vector and $W^{l}$ is a weight matrix. Symbols T and ∥ denote transposition and concatenation operations, respectively. $f_{i}^{m,l}$ and $f_{j}^{m,l}$ denote the embedding vectors of miRNA $m_{i}$ and $m_{j}$ obtained from the *l*th layer. Then, the normalized attention coefficient is calculated using the SoftMax function to make different nodes comparable:


(6) $$\alpha_{ij}=\frac{\exp(e_{ij})}{\sum_{p\in N_{i}}\exp(e_{ip})}$$

where $N_{i}$ represents the set containing all the first-order neighbors of miRNA $m_{i}$. At last, the feature vector $f_{i}^{m,l+1}$ of miRNA $m_{i}$ is obtained by neighborhood aggregation strategy:


(7) $$f_{i}^{m,l+1}=\;\mathrm{LeakyReLU}\left(\sum_{j\in N_{i}}\alpha_{ij}W^{l}f_{j}^{m,l}\right)$$

Through above intra-view graph representation learning, two feature matrices $F^{g}\in R^{17247*128}$, $F^{m}\in R^{4798*128}$ and graph encoders can be obtained, where the feature dimensionality are set as 128 and each encoder contains 2 layers to avoid over-smoothing.

#### 2.3.3 Cross-view contrastive loss

Contrastive learning is primarily designed to learn representation by maximining the agreement between similar samples and minimizing it for dissimilar ones, and it has been widely applied for extracting informative representations from biological data, facilitating various tasks such as structure prediction (Wang *et al.* 2021), drug discovery (Singh *et al.* 2023), sequence analysis (Li *et al.* 2023), and image analysis (Sanchez-Fernandez *et al.* 2023).

Because genes and miRNAs are intertwined to significantly influence cellular function and disease progression, we propose a cross-view contrastive loss $L^{\mathrm{cv}}$ for enhancing the representations of genes and miRNAs by incorporating the critical regulatory mechanism between them. $L^{\mathrm{cv}}$ consists of two parts aiming to refine gene and miRNA expressions respectively and can be formulated as:


(8) $$L^{\mathrm{cv}}={\beta L}^{\mathrm{cv}\_g}+(1-\beta )L^{\mathrm{cv}\_m}$$

$L^{\mathrm{cv}\_g}$ and $L^{\mathrm{cv}\_m}$ are the contrastive losses for genes and miRNAs, β is a parameter controlling the importance of loss computed with different central molecules. Inspired by the effectiveness of InfoNCE loss in contrastive learning (Dwibedi *et al.* 2021), we define$\;L^{\mathrm{cv}\_g}$ and $L^{\mathrm{cv}\_m}$ as:


(9) $$L^{\text{cv}\_g}=\sum_{i=1}^{N^{g}}-\text{log}\;\frac{\sum_{j=1}^{N^{m}}\Pi_{i,j}\;\text{exp}(\mathrm{rel}(f_{i}^{g},f_{j}^{m})/\tau )}{\sum_{k=1}^{N^{m}}\;\text{exp}(\mathrm{rel}(f_{i}^{g},f_{k}^{m})/\tau )}$$

(10) $$L^{\text{cv}\_m}=\sum_{p=1}^{N^{m}}-\text{log}\;\frac{\sum_{q=1}^{N^{g}}\Pi_{p,q}\;\text{exp}(\mathrm{rel}(f_{p}^{m},f_{q}^{g})/\tau )}{\sum_{k=1}^{N^{g}}\;\text{exp}(\mathrm{rel}(f_{p}^{m},f_{k}^{g})/\tau )}$$

where rel(·) represents a correlation function, more specifically, $\mathrm{rel}\left(f_{i}^{g},f_{j}^{m}\right)=\frac{f_{i}^{g}{f_{j}^{m}}^{T}}{\left\|f_{i}^{g}\right\|\left\|f_{j}^{m}\right\|}$ denotes the relevance degree between the *i*th gene and *j*th miRNA, while $f_{i}^{g}$ and $f_{j}^{m}$ are their feature vectors [cf Equations (4) and (7)]. $\Pi (\cdot )\in \left\{0,1\right\}$ is an indicator function, Π(⋅)=1 if the target gene and miRNA are interacted in the network $G^{gm}$, otherwise 0. $N^{g}$ and $N^{m}$ are the numbers of genes and miRNAs, respectively, and τ is a temperature parameter controlling the scale of distribution.

### 2.4. Association pattern joint learning

#### 2.4.1 Disease pair feature extraction

To explore underlying molecular mechanism and obtain meaningful representation of diseases, we construct disease features from both genetic and transcriptional views. The associations between diseases and genes, miRNAs are collected from networks $G^{dg}$ and $G^{dm}$, and the average pooling method is used to obtain feature vectors $f_{i}^{d\_g}$ and $f_{i}^{d\_m}\;$for disease $d_{i}$:


(11) $$f_{i}^{d\_g}=\frac{1}{\left|G_{i}\right|}\sum_{g_{j}\epsilon G_{i}}f_{j}^{g}$$

(12) $$f_{i}^{d\_m}=\frac{1}{\left|M_{i}\right|}\sum_{m_{p}\epsilon M_{i}}f_{p}^{m}$$

where $G_{i}$ and $M_{i}$ are two sets containing the genes and miRNAs associated with disease $d_{i}$, while $f_{j}^{g}$ and $f_{p}^{m}$ are the feature vectors of gene $g_{j}$ and miRNA $m_{p}$.

Different from unsupervised measurement, we attempt to detect complex association pattern between diseases by considering supervised signals. For each disease pair <$d_{i}$, $d_{j}$> in $S_{\mathrm{benchmark}}$ and $S_{\mathrm{independent}}$ [cf Equation (1)], two pair features $h_{i,j}^{d\_g}$ and $h_{i,j}^{d\_m}$ can be obtained by concatenating strategy based on disease feature matrices $F^{d\_g}\in R^{30170*128}$ and $F^{d\_m}\in R^{30170*128}$, where $h_{i,j}^{d\_g}=\left[f_{i}^{d\_g},f_{j}^{d\_g}\right]$ and $h_{i,j}^{d\_m}=\left[f_{i}^{d\_m},f_{j}^{d\_m}\right]$. Furthermore, two multi-layer perceptron networks, each including 256 neurons for the hidden layer and 128 neurons for the output layer, are designed to extract high-level feature matrices $\hat{H^{d\_g}}\in R^{N^{p}*128}$ and $\hat{H^{d\_m}}\in R^{N^{p}*128}$ for disease pairs:


(13) $$\hat{H^{d\_g}}=\sigma (W^{2,g}\sigma (W^{1,g}H^{d\_g}))$$

(14) $$\hat{H^{d\_m}}=\sigma (W^{2,m}\sigma (W^{1,m}H^{d\_m}))$$

where $N^{p}$ represents the number of disease pairs in $S_{\mathrm{benchmark}}$ [cf Equation (1)], $W^{1,g}$, $W^{2,g}$, $W^{1,m}$and $W^{2,m}$ are learnable weight matrices from different fully connected layers, σ is defined as ReLU function. Normalization operation is conducted after each network layer for reducing internal covariate shift and improving stability.

#### 2.4.2 Disease similarity prediction

To capture hidden association pattern between diseases, we design an attentive multi-layer perception network constituting of attentive layer and similarity computation layer. The attentive layer aims to integrate the high-level features of diseases $\hat{H^{d\_g}}$ and $\hat{H^{d\_m}}$ by considering the feature correlation and contribution within different disease pairs. The feature matrix $\bar{H^{d}}$ extracted from attentive layer can be formulated as:


(15) $$\bar{H^{d}}=\sigma (\varphi (W^{\mathrm{cor}}\hat{H^{d}})⊙\hat{H^{d}})$$

where $\hat{H^{d}}\in R^{N^{p}*256}$ is initially constructed by concatenating $\hat{H^{d\_g}}$ and $\hat{H^{d\_m}}$, and $W^{\mathrm{cor}}$ is a learnable feature correlation matrix. ⊙ denotes Hadamard product operation between matrices, σ is defined as ReLU function, and φ is a column-wise normalization operation defined as:


(16) $$\varphi (M_{ij})=\frac{\;\text{exp}(M_{ij})}{\sum_{p=1}^{N^{p}}\;\text{exp}(M_{ip})}$$

$M=W^{\mathrm{cor}}\hat{H^{d}}$ . The feature matrix $\bar{H^{d}}\in R^{N^{p}*256}$ is further fed into a fully connected network layer consisting of 256 neurons, after which a single neuron is used to calculate disease similarity scores. The valuable disease association information is considered as supervision signal for providing critical guidance for model optimization. To accelerate training efficiency, binary cross-entropy with logits loss is used and formulated as:


(17) $$\begin{array}{c} L^{\text{sim}}=-\frac{1}{N^{p}}\sum_{i=1}^{N^{p}}[y_{i}*\;\text{log}(\frac{1}{1+\text{exp}(y_{i}^{\prime })})\\ +(1-y_{i})*\;\text{log}(1-\frac{1}{1+\text{exp}(y_{i}^{\prime })})] \end{array}$$

$y_{i}$ and $y_{i}^{\prime }$ denote the true label and predicted similarity score for the *i*th disease pair.

Overall, the cross-view contrastive learning and association pattern joint learning are horizontally and vertically collaborative to reveal the deep correlation between diseases. The integrated loss function is $L^{\mathrm{all}}=L^{\mathrm{sim}}+L^{\mathrm{cv}}$, and each unknown disease pair can be evaluated by the trained predictor.

### 2.5. Performance evaluation

Four comprehensive indicators including Area Under the Receiver Operating Characteristics Curve (AUC), Area Under the Precision-Recall Curve (AUPR), F1-Score, and Matthews Correlation Coefficient (MCC) are adopted to evaluate the performance of various methods (Zulfiqar *et al.* 2023, Ma *et al.* 2024). F1-Score and MCC are two balanced metrics that take into account precision and recall, as well as true positive and negative rates, and false positive and negative rates, respectively. AUC illustrates the trade-off between sensitivity and specificity while AUPR describes the trade-off between precision and recall at various threshold settings (Hasanin *et al.* 2019, Chen *et al.* 2023b). Besides, three conditional indicators including accuracy (Acc), Sensitivity (Sen) and Precision (Pre) are also used to evaluate different models. It is widely acknowledged that the disease pairs with higher predicted scores tend to be focused more, therefore two additional indicators are used. One is ROC*k* describing the area under Receiver Operating Characteristics Curve (ROC) up to the *k*th false positives (Gribskov and Robinson 1996), while the other measures the number of true positive pairs within top *n* predicted pairs.

## 3 Results and discussion

### 3.1 Parameter analysis

**Figure 2.**
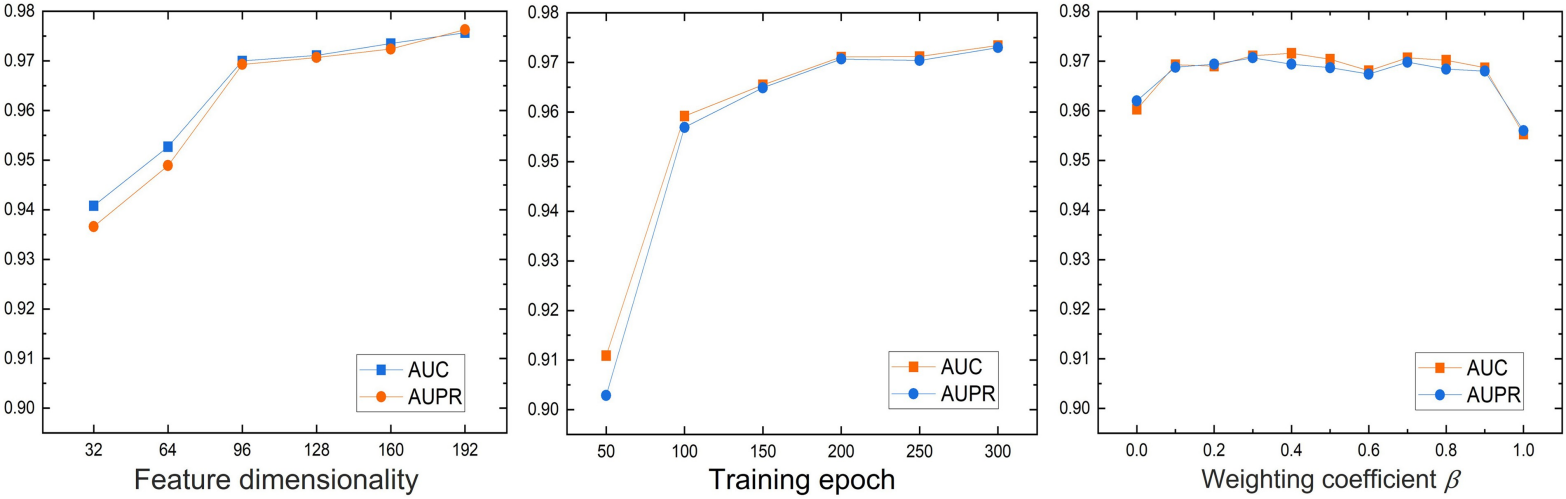
Parameter analysis of DiSMVC. The influence of disease feature dimensionality, training epoch and weighting coefficient *β* on the performance of DiSMVC.

The impact of three important parameters including disease feature dimensionality, training epoch and weighting coefficient *β* [cf Equation (8)] are analyzed. The benchmark dataset $S_{\mathrm{benchmark}}$ is randomly divided into five folds, where four folds are used for training while one remaining fold is used for validating. The parameter analysis experiments are performed by varying one parameter while fixing others. The results are shown in Fig. 2, from which we can see that the performance of DiSMVC initially improves and relatveily stabilizes with the increment of the feature dimensionality and epoch. The impact of the weighted coefficient *β* on the performance of DiSMVC is not significant. However, we can still observe that integrating two contrastive losses for genes and miRNAs can contribute to improving performance. With regard to both prediction accuracy and training time, we set the values of the disease feature dimensionality, training epoch, and weighted coefficient *β* as 128, 200, and 0.3, respectively.

### 3.2 Performance comparison of different methods

**Figure 3.**
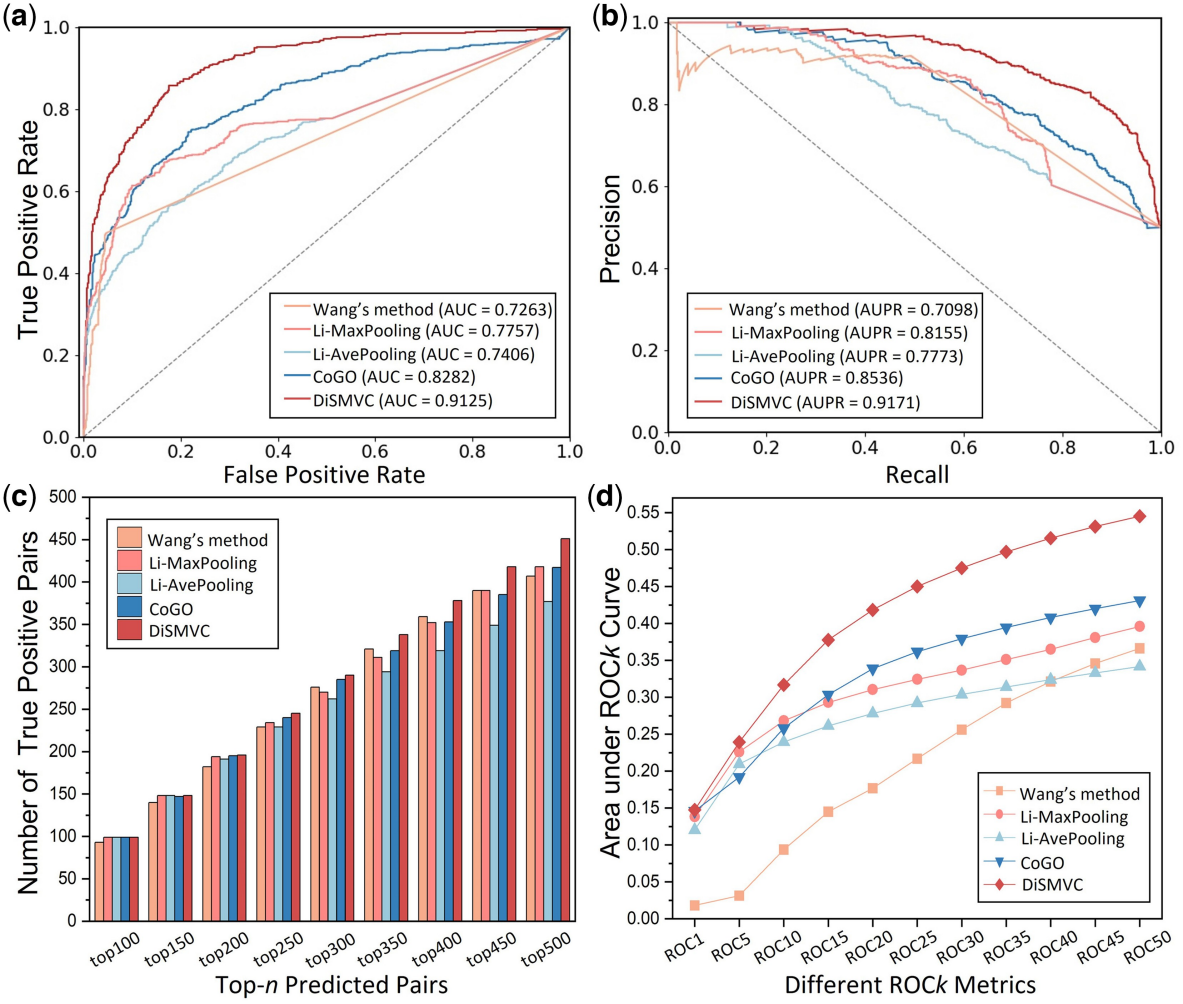
Performance comparison of different methods on $S_{\mathrm{independent}}$. (a) and (b) show the ROC and PR curves with their corresponding AUC and AUPR scores obtained by different methods. (c) shows the numbers of true positive pairs in the top-n pairs predicted by various methods, and (d) shows the ROC*k* scores obtained by different predictors.

To illustrate the effectiveness of DiSMVC, we compare DiSMVC with five state-of-the-art methods, including Wang’s method (Wang *et al.* 2007), Li-MaxPooling (Yang *et al.* 2021), Li-AvePooling (Yang *et al.* 2021), CoGO (Chen *et al.* 2022), and SynerSim (Gao *et al.* 2023). The first four methods measure disease similarity in an unsupervised manner. Wang’s method measures disease similarity based on their Directed Acyclic Graphs (DAGs), where diseases tend to be similar if they share disease semantic terms significantly. Li-MaxPooling, Li-AvePooling, and CoGO are three molecule-based methods, that extract disease features via different graph representation techniques. SynerSim utilizes initial highly sparse disease association network as self-supervised information to guide the association pattern mining. In contrast, DiSMVC introduces additional phenotype-annotated association supervision signals, enabling biological semantics enhancement and label leakage avoidance. Since the source code or web server of SynerSim is not accessible, we further conduct an unbiased performance comparison of DiSMVC with other four unsupervised methods. The performance comparison results are shown in Table 2 and Fig. 3, from which we can see the following: (i) compared to semantic-based method, the molecule-based predictors obtain superior performance in terms of AUC, AUPR, F1-Score and MCC, attributing to their powerful ability of uncovering hidden molecule pathogenic mechanism; (ii) in contrast with the unsupervised measurement, DiSMVC can capture more informative and deeper disease semantic by integrating multiple bio-entity association knowledge and supervision signals. As a result, DiSMVC achieves obviously higher performance than all the competing methods in terms of the comprehensive classification metrics; (iii) Fig. 3c and d shows the number of true positive pairs in the top-*n* pairs and the ROC*k* scores obtained by different predictors. Benefiting from the multi-view graph collaborative learning framework, DiSMVC can also improve the quality of top-ranked results.

**Table 2.** Performance comparison of different methods on $S_{\mathrm{independent}}$

Method	AUC	AUPR	F1-Score	MCC	Acc	Sen	Pre
Wang’s method	0.7263	0.7098	0.6446	0.5068	0.7256	0.4977	0.9144
Li-MaxPooling	0.7757	0.8155	0.7295	0.5108	0.7519	0.6692	0.8018
Li-AvePooling	0.7406	0.7773	0.6915	0.3236	0.6579	0.7669	0.6297
CoGO	0.8282	0.8536	0.7597	0.5265	0.7632	0.7489	0.7709
DiSMVC	0.9125	0.9171	0.8423	0.6800	0.8398	0.8556	0.8294

### 3.3 Feature analysis

**Figure 4.**
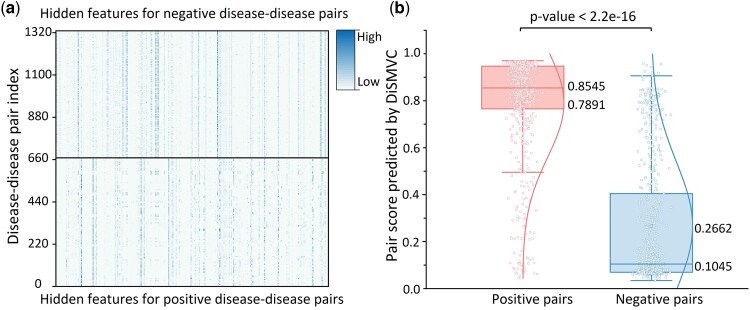
An analysis of features learned by DiSMVC. (a) shows the visualized heatmap of hidden features of disease pairs extracted by DiSMVC. The rows denote the pair index of positive pairs and negative pairs from $S_{\mathrm{independent}}$, and the columns represent the corresponding feature index. (b) shows the similarity score distribution of disease pairs within different types obtained by DiSMVC.

To explore the reason for the performance improvement of DiSMVC for measuring disease similarity, the disease pair features and similarity scores learned by DiSMVC are analyzed and shown in Fig. 4. From Fig. 4 we can see the following: (i) the visualized features of disease-disease pairs within the same type are similar, but the hidden features of positive pairs are obviously different from those of negative pairs, illustrating that DiSMVC can uncover the similar pattern within the same pair group, as well as the specific patterns between different pair groups; (ii) the extracted high-level features further facilitate to the strong discriminative ability of DiSMVC. As a result, the similarity scores predicted by DiSMVC show significant differences between two different pair groups, where most disease pairs in positive group are similar and those in negative group have much lower similarity scores.

### 3.4 Cross-view graph contrastive learning and association pattern joint learning collaboratively enhance prediction ability

DiSMVC is constituted of two crucial modules including cross-view graph contrastive learning and association pattern joint learning. To analyze their contributions to the prediction ability of DiSMVC, we construct seven competing baseline methods and conduct an ablation study.

**Figure 5.**
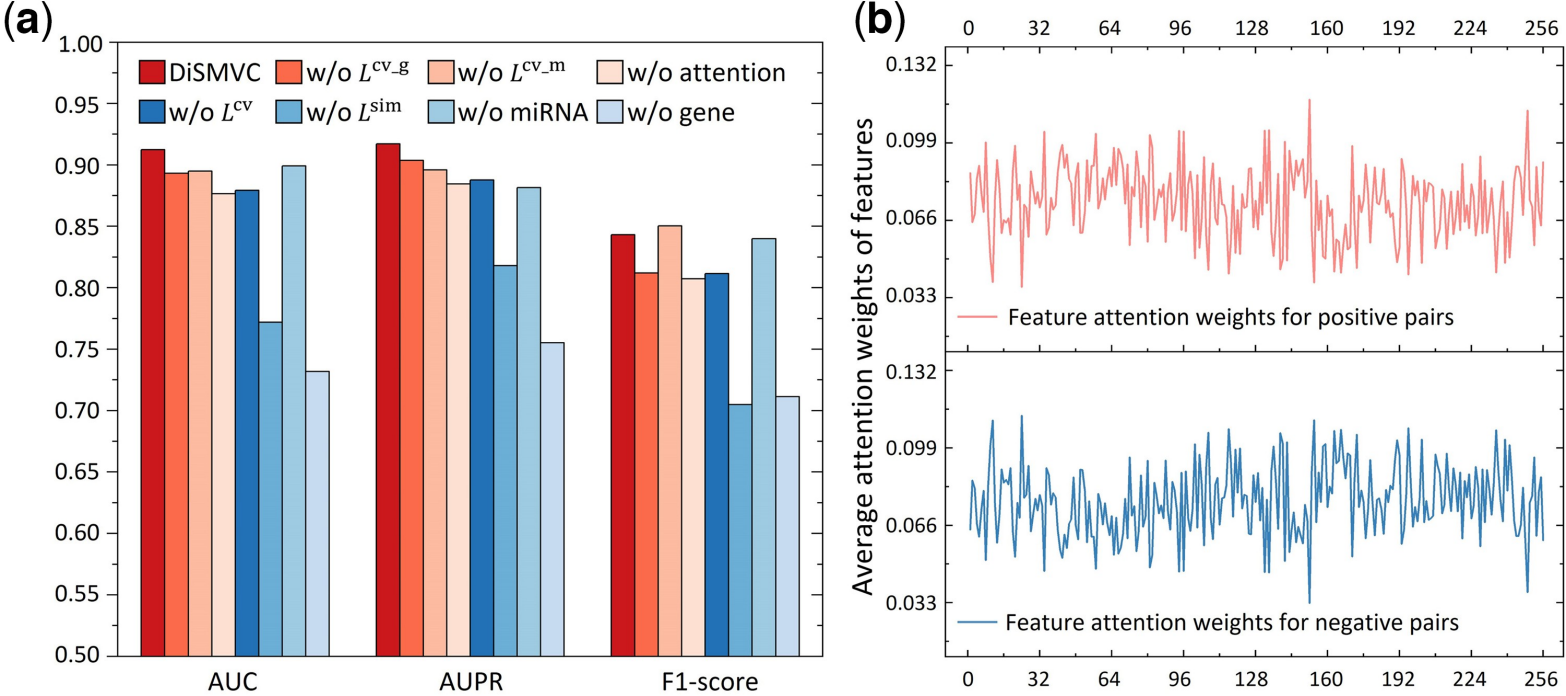
An importance analysis of different modules in DiSMVC. (a) shows the performance comparison of DiSMVC with other seven baseline methods. ‘w/o $L^{\mathrm{cv}\_g}$’, ‘w/o $L^{\mathrm{cv}\_m}$’, and ‘w/o $L^{\mathrm{cv}}$’ are three variants of DiSMVC trained without computing contrastive loss $L^{\mathrm{cv}\_g}$, $L^{\mathrm{cv}\_m}$ or $L^{\mathrm{cv}}$. ‘w/o $L^{\mathrm{sim}}$’ combines cross-view graph contrastive learning and cosine correlation analysis to measure disease similarity. ‘w/o miRNA’ and ‘w/o gene’ are two variant methods where hidden features of genes or miRNAs separately extracted by intra-view graph representation are used to construct disease pair features, and training attentive multi-layer perception to predict disease similarity scores. ‘w/o attention’ integrates cross-view graph contrastive learning and association pattern joint learning without attention mechanism. (b) shows the average feature attention weights obtained by DiSMVC for the disease pairs in $S_{\mathrm{independent}}$.

The variants of DiSMVC are named ‘w/o $L^{\mathrm{cv}\_g}$’, ‘w/o $L^{\mathrm{cv}\_m}$’, ‘w/o $L^{\mathrm{cv}}$’, ‘w/o $L^{\mathrm{sim}}$’, ‘w/o miRNA’, ‘w/o gene’, and ‘w/o attention’, respectively. Their predictive performance along with DiSMVC is shown in Fig. 5, from which we can draw the following conclusions: (i) almost all the variants of DiSMVC achieve inferior performance in terms of AUC, AUPR, and F1-Score, indicating that each module in DiSMVC is important, and cross-view graph contrastive learning and association pattern joint learning collaboratively advance the performance; (ii) different from DiSMVC, ‘w/o $L^{\mathrm{sim}}$’ computes pair score in an unsupervised manner. A considerable performance discrepancy is observed between them, hence proving the significant contribution of phenotypic multimorbidity-based supervision signal in measuring disease similarity; (iii) DiSMVC is superior to ‘w/o miRNA’ and ‘w/o gene’, illustrating the advantages of enriching disease representation by considering underlying molecular mechanisms from both genetic and transcriptional views. Meanwhile, the significant decrease observed in ‘w/o gene’ suggests that genes play a more crucial role in disease association detection compared to miRNA. It is worth noting that ‘w/o attention’ uses concatenate operation to integrate disease embeddings derived from gene and miRNA features, resulting in limited performance improvement. In contrast, DiSMVC integrates features from multiple views by considering their different importance via attention layer, as illustrated in Fig. 5b, enabling effective fusion and strong prediction ability.

### 3.5 Case study

To illustrate the validity of DiSMVC for measuring disease similarity, we conduct case study on the interpretability of top disease pairs predicted by DiSMVC. The 10th revision of International Classification of Diseases (ICD-10) is globally recognized and widely used for categorizing and coding various diseases, therefore, we analyze the predictive results along with ICD-10 category. All the candidate disease pairs with ICD-10 code in $S_{\mathrm{independent}}$ are predicted and further ranked in descending order of pair scores.

**Figure 6.**
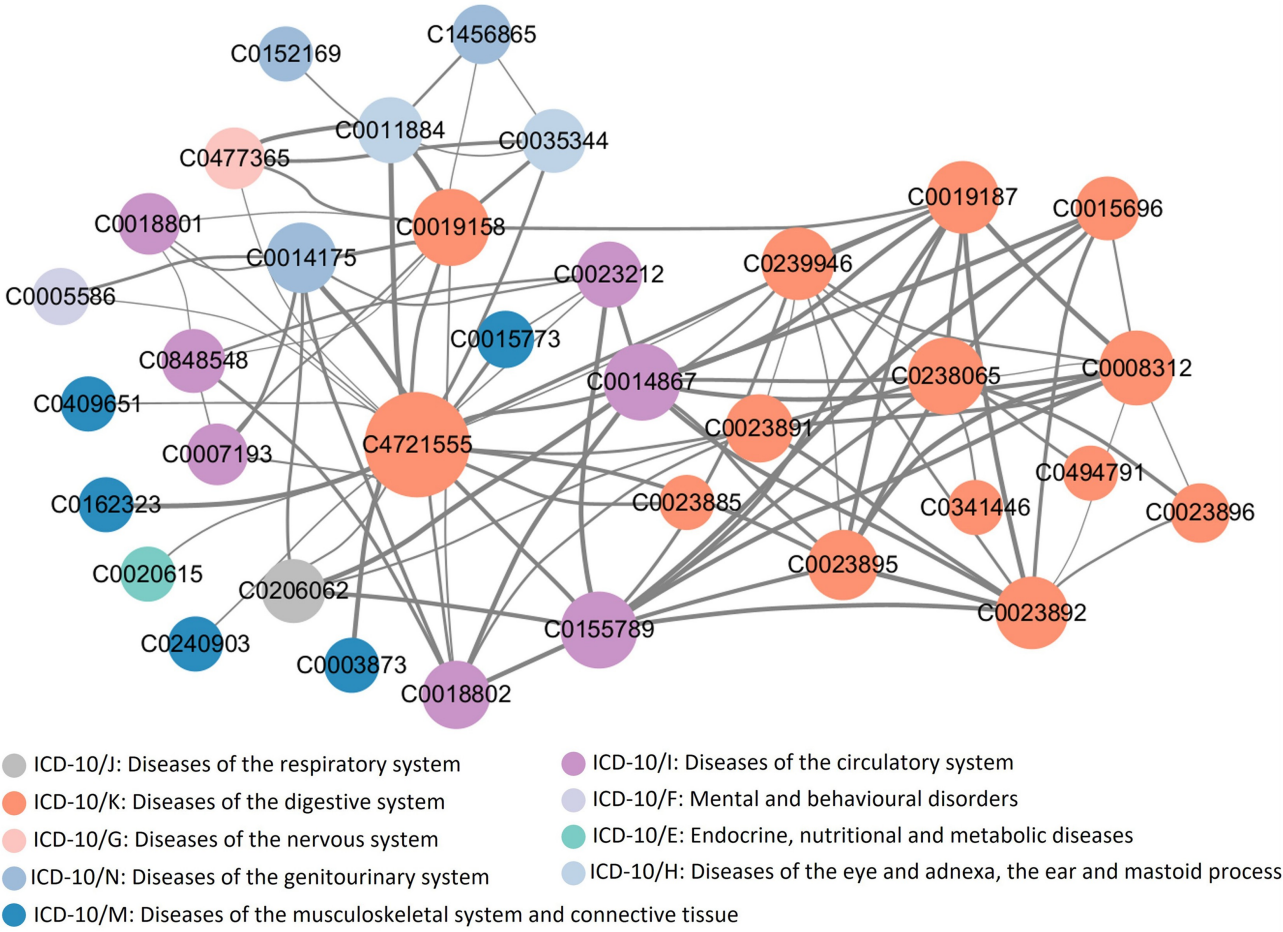
The network of top 100 disease pairs predicted by DiSMVC. The diseases with UMLS concept unique identifier are shown in circles, where the size illustrates the number of related diseases, and the color represents disease types classified based on ICD-10 version. The thickness of edges denotes the different pair scores between diseases predicted by DiSMVC, where thicker edges indicate closer relations.

The network of top 100 disease pairs predicted by DiSMVC is shown in Fig. 6, from which we can see that diseases belonging to the ‘ICD-10/K: Disease of digestive system’ or ‘ICD-10/I: Disease of the circulatory system’ are more likely to be in closed proximity, indicating that the disease relationship predicted by DiSMVC to some extent conforms to the ICD-10 classification. In addition, DiSMVC can also identify disease associations from different categories via exploring underlying molecular mechanisms. For example, the autoimmune hepatitis labeled as C4721555 belonging to ICD-10/K shows close correlation with other four types of diseases including C0162323, C0011884, C0014175, and C0155789.

**Figure 7.**
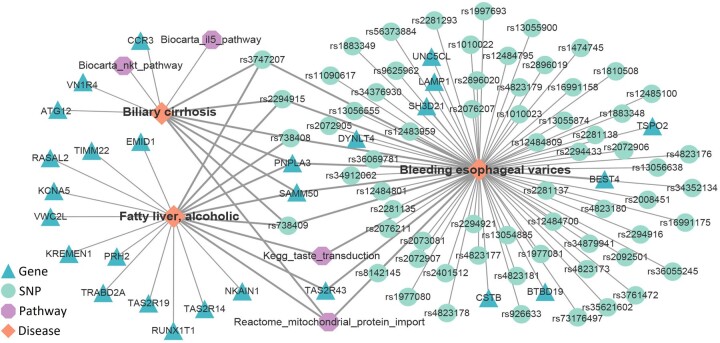
The network of bio-entity associations for three representative diseases. Three diseases are shown in rhombuses, and their associated genes, SNPs, and pathways are shown in triangles, circles, and octagons, respectively

To further investigate the molecular interpretability of predicted disease pairs, the top 10 disease pairs are further analyzed and shown in Table 3. We can observe that seven disease pairs can be supported by experimental literatures in PubMed, where most diseases are accompanied by common molecule or biological process abnormity. For examples, ‘Bleeding esophageal varices’ is a circulatory system disease and ‘Fatty liver, alcoholic’ belongs to the subtype of digestive system disease. Long-term alcoholic fatty liver is one of the common causes of bleeding esophageal varices. Although the two diseases originate from different human systems, they are mediated by two important biological processes including ‘Mitochondrial protein import’ in Reactome (Fabregat *et al.* 2018) and ‘Taste transduction’ in KEGG (Kanehisa and Goto 2000), and significantly associated with PNPLA3 gene and four SNPs including rs738408, rs2294915, rs3747207, and rs738409. Besides, it is reported that cholestasis is one of the key pathogenic factors of alcoholic liver disease. The predicted disease pair of ‘Fatty liver, alcoholic’ and ‘Biliary cirrhosis’ are mediated by overlap genes, SNPs, and pathways as shown in Fig. 7.

**Table 3.** Top 10 disease pairs predicted by DiSMVC.

Rank	Disease	Disease	Evidence
1	Bleeding esophageal varices	Fatty liver, alcoholic	PMID: 34225788
2	Fatty liver, alcoholic	Biliary cirrhosis	PMID: 34225788
3	Diabetic retinopathy	Autoimmune hepatitis	PMID: 30625487
4	Endometriosis	Autoimmune hepatitis	Unconfirmed
5	Liver diseases	Biliary cirrhosis	PMID: 34225788
6	Esophageal varices	Fatty Liver, alcoholic	PMID: 34225788
7	Polyarthritis	Autoimmune hepatitis	PMID: 34025065
8	Esophageal varices	Interstitial pulmonary fibrosis	Unconfirmed
9	Hepatitis, alcoholic	Biliary cirrhosis	PMID: 34225788
10	Bleeding esophageal varices	Left-sided heart failure	Unconfirmed

Overall, DiSMVC can identify similar diseases that are molecularly interpretable and provide guidance for understanding the pathological mechanisms of diseases.

## 4 Conclusion

In this study, we propose a new computational predictor named DiSMVC to measure disease similarity. Compared with other competing methods, it has the following advantages: (i) the graph collaborative learning framework of DiSMVC is able to integrate multiple aspects of disease-related information including bio-entity association at micro-level and phenotype-based multimorbidity at macro-level, showing promising performance and molecular interpretability in measuring disease similarity; (ii) the high-level molecular interaction features related to diseases are extracted and refined through cross-view contrastive learning, based on which more informative disease representation can be obtained from both genetic and transcriptional perspectives; (iii) different from other existing methods, DiSMVC measures disease similarity in a supervised manner by incorporating priori disease association knowledge, so as to capture strongly discriminative association patterns.

There is still potential for further improvement in the study. The occurrence and development of diseases is an extremely complex process that is influenced by various biological and environmental factors. Therefore, considering more disease related bio-entities such as proteins, metabolites and microbes is beneficial to enhance the richness and comprehensiveness of disease representation. We use similar, comorbid conditions as positive labels, leading to limitation in detecting biologically similar diseases that share underlying mechanisms but may not necessarily be comorbid. Incorporating auxiliary supervised signals from DisGeNET can help capture biological similarities between diseases. Finally, the integration of additional knowledge inevitably introduces noise. More high-quality data sources or advanced heterogeneous network learning techniques are expected to extract meaningful information while mitigate noise interference.

## Supplementary Material

btae306_Supplementary_Data
