# Supplementary material for: DiSMVC: a multi-view graph collaborative learning framework for measuring disease similarity
Source: Bioinformatics. 2024 May 7;40(5):btae306. doi: 10.1093/bioinformatics/btae306 (PMC11256965; doi:10.1093/bioinformatics/btae306)
Supplement: btae306_Supplementary_Data [file btae306_supplementary_data.docx]

Supplementary Material

We have randomly sampled data from the benchmark dataset in different proportions relative to the number of samples in the independent testing set to construct the training set. Then, we investigated the impact of different train:test sampling ratios on the predictive performance of DiSMVC. The results are shown in **Figure S1**, from which we can see that with the increment of training samples, DiSMVC can capture more disease similarity patterns, resulting in gradual improvement in predictive performance. Consequently, we utilized all the samples from the benchmark dataset to train the final model.


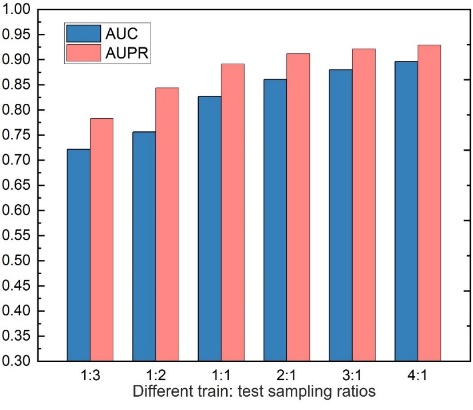


**Figure S1.** Impact of different train:test sampling ratios on the predictive performance of DiSMVC.

The features of disease pairs in the independent testing set were extracted using different methods. The T-Distributed Stochastic Neighbor Embedding (T-SNE) algorithm (Belkina et al. 2019) was utilized to reduce the dimensionality of each disease pair feature. The T-SNE visualization results are shown in **Figure S2**, from which we can see that the disease pair features learned by DiSMVC exhibit superior discriminative ability compared to other competing methods. This further illustrates the advantage of incorporating supervised signals for detecting disease similarity patterns.


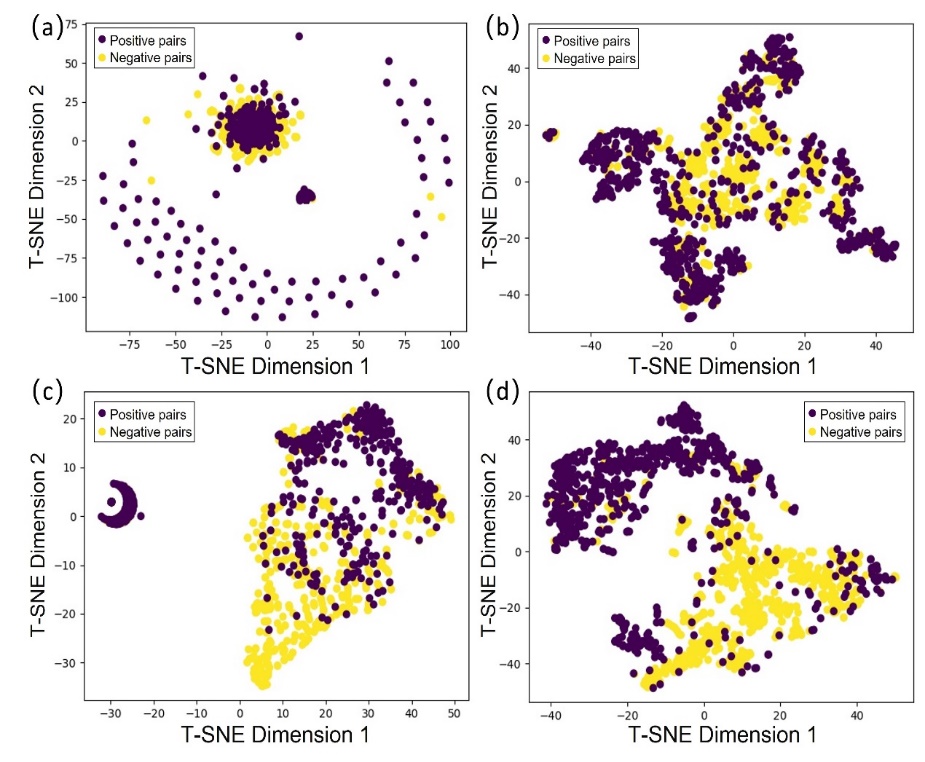


**Figure S2.** Visualization of disease pair features extracted by different methods. (a)-(d) show the T-SNE visualization of disease pair features extracted by Li-AvePooling (Yang et al. 2021), CoGO (Chen et al. 2022), Li-MaxPooling (Yang et al. 2021) and DiSMVC, respectively.

**References**

Belkina, Anna C, et al. (2019), Automated optimized parameters for T-distributed stochastic neighbor embedding improve visualization and analysis of large datasets, *Nature communications,* **10** (1), 5415.

Chen, Y. H., et al. (2022), CoGO: a contrastive learning framework to predict disease similarity based on gene network and ontology structure, *Bioinformatics,* **38** (18), 4380-86.

Yang, L., Wang, K. Q., and Wang, G. H. (2021), Evaluating disease similarity based on gene network reconstruction and representation, *Bioinformatics,* **37** (20), 3579-87.
